# *Hoxb3* Regulates *Jag1* Expression in Pharyngeal Epithelium and Affects Interaction With Neural Crest Cells

**DOI:** 10.3389/fphys.2020.612230

**Published:** 2021-01-11

**Authors:** Haoran Zhang, Junjie Xie, Karl Kam Hei So, Ka Kui Tong, Jearn Jang Sae-Pang, Li Wang, Sze Lan Tsang, Wood Yee Chan, Elaine Yee Man Wong, Mai Har Sham

**Affiliations:** ^1^School of Biomedical Sciences, LKS Faculty of Medicine, The University of Hong Kong, Pokfulam, Hong Kong; ^2^School of Biomedical Sciences, The Chinese University of Hong Kong, Shatin, Hong Kong

**Keywords:** *Hoxb3*, *JAG1*, pharyngeal arch, epibranchial placodes, pharyngeal epithelium, cranial neural crest, craniofacial development

## Abstract

Craniofacial morphogenesis depends on proper migration of neural crest cells and their interactions with placodes and other cell types. *Hox* genes provide positional information and are important in patterning the neural crest and pharyngeal arches (PAs) for coordinated formation of craniofacial structures. *Hox* genes are expressed in the surface ectoderm and epibranchial placodes, their roles in the pharyngeal epithelium and their downstream targets in regulating PA morphogenesis have not been established. We altered the *Hox* code in the pharyngeal region of the *Hoxb3^*Tg*/+^* mutant, in which *Hoxb3* is driven to ectopically expressed in *Hoxb2* domain in the second pharyngeal arch (PA2). In the transgenic mutant, ectopic *Hoxb3* expression was restricted to the surface ectoderm, including the proximal epibranchial placodal region and the distal pharyngeal epithelium. The *Hoxb3^*Tg*/+^* mutants displayed hypoplasia of PA2, multiple neural crest-derived facial skeletal and nerve defects. Interestingly, we found that in the *Hoxb3^*Tg*/+^* mutant, expression of the Notch ligand *Jag1* was specifically up-regulated in the ectodermal pharyngeal epithelial cells of PA2. By molecular experiments, we demonstrated that Hoxb3 could bind to an upstream genomic site S2 and directly regulate *Jag1* expression. In the *Hoxb3*^*Tg/+*^ mutant, elevated expression of *Jag1* in the pharyngeal epithelium led to abnormal cellular interaction and deficiency of neural crest cells migrating into PA2. In summary, we showed that *Hoxb3* regulates Jag1 expression and proposed a model of pharyngeal epithelium and neural crest interaction during pharyngeal arch development.

## Introduction

An important phase of mammalian craniofacial development is the formation of the transient pharyngeal arch (PA) structures. These PAs are comprised of an outer surface ectoderm, an inner covering of endoderm, a mesenchymal core, and the cranial neural crest-derived ectomesenchyme. The coordinated development of the different embryonic components give rise to the pharynx, the jaw, the ear and the face. Dysregulation of PA development can lead to many human congenital craniofacial malformations.

The cranial neural crest cells which delaminate from the dorsal region of hindbrain rhombomeres have important structural roles in craniofacial morphogenesis. Neural crest cells originating from different anteroposterior levels of the hindbrain rhombomeres are marked by combinations of *Hox* genes which specify their identity. Molecular analysis have shown that regulation of *Hox* expression in the hindbrain rhombomeres and in the neural crest could be independently controlled by separate *cis*-acting regulatory elements and *trans*-acting factors (Frasch et al., 1995; Nonchev et al., 1996; Maconochie et al., 1999; Trainor and Krumlauf, 2000). The neural crest cells arising from distinct rhombomere locations are not pre-patterned, but remain plastic, and can respond to signals in the environment they migrate to Trainor and Krumlauf (2000) and Le Douarin et al. (2004). In the PAs, neural crest cells respond to signals from the pharyngeal surface ectoderm and the endoderm in determining their cell fate (Graham et al., 2005). The neural crest cells give rise to cranial ganglia and nerves, muscle and facial bones and cartilages (Frisdal and Trainor, 2014). Defective migration, survival, proliferation, or differentiation of the cranial neural crest cells lead to multiple craniofacial abnormalities (Santagati and Rijli, 2003; Minoux and Rijli, 2010). More importantly, PAs can be formed and their antero-posterior and proximo-distal axes maintained in the absence of neural crest cells, indicating the important roles of pharyngeal epithelium and other cell types during PA development (Veitch et al., 1999; Gavalas et al., 2001; Trainor and Krumlauf, 2001).

The ectodermal pharyngeal epithelium covering the proximal and distal regions of the PAs are developmentally distinct. In the proximal PA region, epibranchial placodal cells not only give rise to neurogenic cranial ganglia, but also non-neuronal epithelial cells which play essential roles in pharyngeal segmentation (Zhang et al., 2017). The invagination of the proximal non-neuronal placode-derived epithelium and the outgrowth of the pharyngeal endoderm form the segmental plates, the contact points of the epithelial layers give rise to the pharyngeal clefts and pouches (Graham and Smith, 2001; Graham, 2003; Kulesa et al., 2010). Defective pharyngeal segmentation as displayed in the *Sox3* (Rizzoti and Lovell-Badge, 2007) and *Eya1* mutants (Xu et al., 2002; Zhang et al., 2017) would lead to abnormal PA development. Around the pharyngeal clefts, the non-neuronal epibranchial placodal epithelial cells express various signaling factors including Notch and Fgfs that are required for PA morphogenesis (Trokovic et al., 2005; Wang et al., 2020). The roles of the ectodermal pharyngeal epithelium in interacting with neural crest and other cell types in the developing PAs are not well understood.

Notch signaling factors are required for cranial ganglia neurogenesis and craniofacial morphogenesis (Wakeham et al., 1997; Jayasena et al., 2008; Lassiter et al., 2010; Lassiter et al., 2014). Mutations of the Notch ligand *JAG1* caused Alagille syndrome with craniofacial defects in human patients (Li et al., 1997; Kamath et al., 2003). *Jag1b* mutation in zebrafish led to mis-patterning of pharyngeal arch derived skeletons (Barske et al., 2016), while *Jag2* mutant mice displayed cleft palate (Vieira et al., 2005; Casey et al., 2006). Neural crest-specific knockout of *Jag1* or *Notch2* resulted in middle ear bone malformation (Humphreys et al., 2012; Teng et al., 2017). Knockout of *Rbpj*, a transcriptional cofactor of Notch, in neural crest cells led to shortened mandible formation (Mead and Yutzey, 2012). Deletion of Notch signaling target *Hey1* led to deformed pharyngeal arch arteries (Fujita et al., 2016). Interestingly, in *Presenilin1/2* null mutant mice which deficient in γ-secretase, the PA2 was absent (Donoviel et al., 1999). The *Maml1^–/–^;Maml3^–/–^* mutant embryos also displayed hypoplastic or even no PA2, indicating that morphogenesis of PA2 is particularly sensitive to Notch signaling activity (Oyama et al., 2011). The underlying cellular and molecular mechanisms for Notch signaling functions in the development of PAs remain elusive. In our previous studies, we showed that Notch1 intracellular domain (N1-ICD) was required in the epibranchial placodal epithelium in the proximal PAs to control cell fate commitment of neuronal and non-neuronal epibranchial placodal cells (Zhang et al., 2017). Moreover, Notch receptor signaling controlled rostral-caudal patterning of the non-neuronal placodal epithelial cells around the pharyngeal clefts, indicating important roles of Notch signaling in epithelial cell fate commitment and differentiation (Wang et al., 2020).

Combinatorial expression of *Hox* genes in hindbrain rhombomeres, neural crest and pharyngeal surface ectoderm are known to convey positional information in the PAs (Trainor and Krumlauf, 2001; Santagati and Rijli, 2003). Loss-of-function mouse mutant analyses have shown that *Hox* genes of the first three paralogous groups are required for the development of neural crest derivatives in the pharyngeal region. Mutations of *Hoxa* genes would lead to neural crest defects and malformation of craniofacial structures. Inactivation of *Hoxa2* resulted in homeotic transformation of PA2 components into PA1 (Rijli et al., 1993). *Hoxa1* mutation led to deletion of rhombomere 5 and severe reduction of r4, the mutant displayed hypoplasia of PA2 as a result of neural crest deficiency (Chisaka et al., 1992). *Hoxa3* mutants showed deformed neural crest derivatives of PA3 including the hyoid and thyroid cartilage (Chojnowski et al., 2016) and endoderm defects (Su et al., 2001; Kameda et al., 2004). Mutations of *Hoxb* genes led to neurogenic phenotypes. For instance, although double knockout of *Hoxa1* and *Hoxb1* in mice exacerbated the phenotypes of *Hoxa1* mutant and resulted in hypoplasia of PA2 and malformation of middle ear bones (Gavalas et al., 1998), *Hoxb1* mutant embryos did not exhibit any defects in neural crest-derived tissues of PA2, but displayed abnormal neuronal identity in hindbrain r4 (Goddard et al., 1996; Studer et al., 1996). *Hoxb2* mutant mice displayed retracted lower lip and mild craniofacial features (Barrow and Capecchi, 1996), *Hoxb3* null mutant had no craniofacial abnormalities (Manley and Capecchi, 1998). The specific functions of *Hox* genes in the different cell types of the developing PA are not entirely known.

Insights on the roles of *Hox* genes in cranial neural crest development have also been obtained by gain-of-function analysis. Cranial neural crest cells rostral to rhombomere 2 do not express *Hox* genes, and only *Hox*-free neural crest cells are capable of generating facial skeletal components (Couly et al., 1998; Le Douarin et al., 2004). Gain-of-function experiments by electroporation of *Hoxa2* into the *Hox*-free neural crest domain of chick embryos led to absence of lower jaw and frontonasal structures; defective formation of PA1 derived skeletal structures were also observed in ectopic *Hoxa3* and *Hoxb4* expression experiments (Creuzet et al., 2002). Heterotopic transplantation experiments with fragments of neural folds have demonstrated that *Hox*-positive neural crest cells from the posterior region are incapable to replace *Hox*-free neural crest cells. These studies suggest that cross-talks among *Hox* genes, neural crest cells and extrinsic signals in the pharyngeal region, including ectodermal and endodermal derived factors, are required for the formation neural crest derived structures (Couly et al., 2002; Creuzet et al., 2002; Le Douarin et al., 2004; Minoux and Rijli, 2010).

We have previously generated a gain-of-function *Hoxb3* transgenic mutant *Hoxb3*^*Tg*^ and shown that *Hoxb3* could transcriptionally suppress *Hoxb1* in the hindbrain and maintain anterior-posterior identity of rhombomere 4 and 5 (Wong et al., 2011). Using the *Hoxb2-r4* enhancer element (Maconochie et al., 1997; Szeto et al., 2009), we ectopically expressed *Hoxb3* to *Hoxb2* domains, including hindbrain rhombomere 4 and PA2 in *Hoxb3^*Tg*/+^*, and examined the effect of altering the expression of *Hox* code in PA development. Although *Hoxb3* or *Hoxb2* null mutants had no or mild craniofacial defects, the *Hoxb3*^*Tg/+*^ transgenic mutants displayed multiple neural crest-derived abnormalities. We found that ectopic expression of *Hoxb3* was restricted to the pharyngeal epithelium in the *Hoxb3^*Tg*/+^* mutant, allowing the study of interaction between pharyngeal epithelium and migrating neural crest cells. Interestingly, *Jag1* was ectopically expressed in the ectodermal epithelium of PA2, in cells which co-expressed *Hoxb3*. We showed that *Hoxb3* could transcriptionally regulate *Jag1* expression during PA development. Migration of neural crest cells into the PAs was affected, leading to neural crest deficiency and craniofacial defects at later stages. Our results suggest that *Hoxb3* positively regulates the expression of *Jag1* in the pharyngeal epithelial cells and affects the colonization and maintenance of neural crest cells in the developing PAs.

## Materials and Methods

### Experimental Animals

The mouse lines used in this study include *Hoxb3^*Tg*/+^* [*Hoxb3^*Tg*2^* in Wong et al. (2011)], *B2-Cre* [*Hoxb2-r4-Cre* in Szeto et al. (2009)], *Wnt1-Cre* (Chai et al., 2000), and *Z/EG* (Novak et al., 2000). The *Hoxb3*^*Tg/+*^ transgenic mice were maintained in FVB genetic background, other mouse lines were maintained in C57BL/6N background. All animals were housed at the Center for Comparative Medicine Research at the University of Hong Kong. The animal experiments were approved by the University of Hong Kong Committee on the Use of Live Animals for Teaching and Research (CULATR No. 4357–17 and 4588–18).

### Riboprobe Labeling

Plasmids with target cDNA sequence including *Hoxa2* (Nonchev et al., 1996), *Hoxb2* (nucleotide 41–1114 of mRNA), *Hoxb3* (Wilkinson et al., 1989), *Jag1* (Jones et al., 2000) were cloned, linearized (restriction enzymes used were summarized in Table 1) and purified. RNA probes were prepared using 1 μg of purified DNA for reverse transcription and digoxigenin (DIG) labeling (Roche, 11277073910), synthesized RNA probe was precipitated in ethanol, dissolved in DEPC-treated water, and stored at -80°C.

**TABLE 1:** Enzymes used for riboprobe labeling.

**Antisense Probe**	**Restriction enzyme for anti-sense probe**	**RNA polymerase for RNA transcription**	**References**
*Hoxa2*	*Eco*RI	T7	Nonchev et al., 1996
*Hoxb2*	*Bam*HI	T3	This study
*Hoxb3*	*Bam*HI	T3	Wilkinson et al., 1989
*Jag1*	*Eco*RI	T3	Jones et al., 2000

### Whole-Mount RNA *in situ* Hybridization

Embryos were harvested, fixed in 4% paraformaldehyde (PFA) at 4°C overnight, dehydrated in a series of methanol/PBST (Phosphate-buffered saline with 0.1% Tween-20) solutions and stored in absolute methanol at -20°C. Before *in situ* hybridization, samples were rehydrated with gradients of PBST series, treated with 1 μg/ml proteinase K, then post-fixed in 4% PFA, 0.2% glutaraldehyde in PBS. Embryos were then incubated with hybridization mix (50% formamide, 1.3x SSC, 50 μm EDTA, 50 μg/ml, 0.2% Tween20, 0.5% CHAPS, and 100 μg/ml heparin) at 65°C for 2 h, then incubated with the DIG-labeled RNA probe in hybridization mix at 65°C overnight. After hybridization, samples were washed with hybridization mix in maleic acid buffer with Tween-20 (MABT), blocked with 10% blocking reagent (Roche, 11096176001) and 20% heat-inactivated horse serum, and then incubated in 1:2000 alkaline phosphatase conjugated anti-DIG antibody (Roche, 11093274910) in the same blocking buffer at 4°C. After washing with MABT for 24 h, embryos were immersed in BM purple (Roche, 11442074001) for color development. Reaction was stopped by washing with PBST and post-fixed in 4% PFA.

### Immunostaining and Antibodies

For whole mount immunostaining, E10.5 mouse embryos were harvested and fixed in 4% PFA for 1.5 h, followed by inactivation of endogenous peroxidase in 0.05% peroxide in PBS at 4°C overnight. Embryos were then blocked in PBS-TS (10% heat inactivated serum, 1% Triton X-100 in PBS) for 3 h at 4°C, incubated with 2H3 anti-neurofilament primary antibody (1:100, Developmental Studies Hybridoma Bank) at 4°C for 3 days. Embryos were then incubated with peroxidase-conjugated rabbit anti-mouse secondary antibody (1:200, Dako) and subjected to 4-chloro-1-napthol (Sigma) color development. The reaction was stopped by washes of 30% ethanol.

For section immunostaining, 10 μm thick cryo-sectioned samples were blocked with 10% heat-inactivated horse serum in PBS for 1 h. The sections were then incubated with primary antibodies including GFP [1:500, Rockland Immunochemicals, 600-101-215), Pax8 (1:500, Proteintech, 10336-1-AP), and Jag1 (1:300, Developmental Studies Hybridoma Bank, TS1.15H)] and E-cadherin (1:500, Cell signaling, 24E10) at 4°C overnight. After three washes of PBS, Alexa Fluor 488- or 594-conjugated secondary antibodies (1:500, Life technologies) were applied. DAPI (Sigma-Aldrich, D9542) was used as a counterstain for the nucleus. Sections were washed with PBS then mounted with mounting medium (Vectashield, Vector Laboratories).

### Scanning Electron Microscopy

Embryos were fixed in 2.5% glutaraldehyde for 4 h and then in 1% osmium tetroxide for 1 h, dehydrated in graded ethanol and critical-point dried with transition fluid liquid carbon dioxide in a Ladd critical point dryer. Gold-palladium-coated specimens were examined with a JEOL JSM-6301FE scanning electron microscope operated at 5 KV.

### Skeletal Preparations

Newborn mice were fixed in 95% ethanol overnight. After skin removal, the whole-mount skeletons were incubated in 0.015% Alcian Blue and 0.005% Alizarin Red stain diluted in 5% acetic acid in 70% ethanol for 1 week (Mcleod, 1980). The preparations were cleared in 20% glycerol, 1% potassium hydroxide, and then transferred to 50% glycerol/ethanol for photography.

### Bioinformatics Analysis of Hoxb3 Binding Sites

The matrix of Hoxb3 binding sequence (PH0058.1) was obtained from the JASPAR database. Hoxb3 binding motif was scanned along 100 kb upstream of *Jag1* locus using Find Individual Motif Occurrences v5.0.5 (FIMO) with *p* < 0.0005 as cutoff. The genomic loci were further analyzed for sequence conservation among human, chicken, lizard and Xenopus with PhyloP score higher than 1. Genomic regions were further aligned with open chromatin regions in developing mouse embryo (E11.5) ATAC-seq data with following accession code: ENCSR150RMQ (facial prominence), ENCSR273UFV (forebrain), ENCSR382RUC (midbrain), ENCSR012YAB (hindbrain), and ENCSR282YTE (neural tube).

### P19 Cell Culture

P19 mouse embryonal carcinoma cells were cultured on gelatin-coated (0.1%) tissue culture dishes in Dulbecco’s Modified Eagle Medium (DMEM) medium (Gibco) supplemented with 10% FBS. Retinoic acid (RA) treatment (10^–8^ M) of P19 cells for activation of hindbrain *Hox* genes expression were performed as previously described (Okada et al., 2004; Wong et al., 2011). Using 10^–8^ M RA, expression of anterior *Hox* genes including Hoxb3 would be activated endogenously.

### Quantitative Real-Time PCR

Total RNA samples were extracted according to TRIzol manufacturer’s protocol from RA treated and control P19 cells. cDNA samples were generated from total extracted RNA using Superscript III (ThermoFisher) and polymerase chain reaction (PCR) performed with SYBR Green Premix Ex Taq II (TaKaRa) using the StepOnePlus Real-Time PCR system. Primers for *Hoxb3*: forward: 5′-GCAGA AAGCC ACCTA CTACG-3′, reverse: 5′-CCATT GAGCT CCTTG CTCTT-3′; for *Jag1*: forward: 5′-AACAC AGGGA TTGCC CACTT-3′, reverse: 5′-TGTTG CAATC AGGAC CCATC-3′; for Gapdh: forward: 5′-TTCAC CACCA TGGAG AAGGC-3′; reverse: 5′-GGCAT GGACT GTGGT CATGA-3′. Relative expression of *Hoxb3* and *Jag1* were normalized with *Gapdh* expression. All the experiments were performed in triplicates.

### Chromatin Immunoprecipitation Assay

Chromatin immunoprecipitation (ChIP) assay (Hu and Rosenblum, 2005) was performed with the following modifications. For RA-treated P19 cells, 1 × 10^6^ cells were harvested for each assay. For *in vivo* ChIP, 4 litters of E9.5 wildtype whole embryos were harvested in PBS, fixed in 1% formaldehyde for 20 min at 25°C, and then disintegrated with RIPA buffer. The cross-linked material was sonicated to 200–1,000 bp fragments (Vibracell sonicator; seven times for 10 s at 40% output), anti-Hoxb3 antibody (Santa Cruz, C-20, and sc-17169) or normal rabbit IgG were then used to pull down the chromatin. PCR amplifications were performed using the following primers: for S1: forward: 5′-AGTCA TCGTC TGCTG CCTTT-3′, reverse: 5′-GCAAC GAATT CATTC AGCAA-3′; for S2: forward: 5′-TTTTA GCCCC TGCTT GCTTA-3′, reverse: 5′-TTGGA GAACA GCCCT CATTT-3′; for S3: forward: 5′-CCTAA CCCCT TTCCC ATCAT-3′, reverse: 5′-TTCTT GTTTG GGCTT GCTCT-3′; for B3ARE: forward: 5′-GTAGG TGTGT GGGCA GAGGT-3′, and reverse: 5′-CTGAG TGGAG GATGG GTTGT-3′. The experiments were performed with 3 biological replicates and 3 technical replicates.

### *In vitro* and *ex vivo* Luciferase Activity Assays

For the *in vitro* transactivation experiments, *pCIG-Hoxb3* cDNA (Yau et al., 2002) or *pCIG* control expression vector was co-electroporated with Firefly and Renilla luciferase reporters into human embryonic kidney cells 293T. The luciferase reporter constructs were *pGL3-Jag1-S2* which contained a 400 bp fragment (*Jag1* genomic DNA, mm10 chr2: 136979005-136979405) on the *Jag1* locus with the Hoxb3 binding site S2 CTGTAATTAACT, or *pGL3-Jag1-mS2* which contained a mutated Hoxb3 binding site mS2 CTGGCCGGCACT, or *pGL3* control luciferase vector. After 24 h, the cells were processed for luciferase assay as previously described (Dessaud et al., 2007; Wong et al., 2011). Briefly, cells were homogenized in lysis buffer on ice, Firefly and Renilla luciferase activities were measured with the Dual Luciferase Reporter Assay System (Promega).

For the *ex vivo* luciferase activity assay, *pCIG-Hoxb3* cDNA or *pCIG* control vector, together with *pGL3-Jag1-S2, pGL3-Jag1-mS2*, or control luciferase reporter construct, and Renilla luciferase reporter, were electroporated into the hindbrain region of E9.0 mouse embryos. After cultured for 24 h, the pharyngeal portion (Figure 4D) of the embryos were homogenized in lysis buffer on ice, Firefly and Renilla luciferase activities were measured as above.

All the experiments were performed with 3 biological replicates and 3 technical replicates.

### Statistical Test

Statistical comparison of two groups was performed by Student’s *t*-test, where n number represented biological replicates using the same experimental condition. Error bars indicated standard error of the mean (SEM).

## Results

### Expression of *Hox* Genes in Pharyngeal Ectoderm and Epibranchial Placodal Cells

**FIGURE 1:**
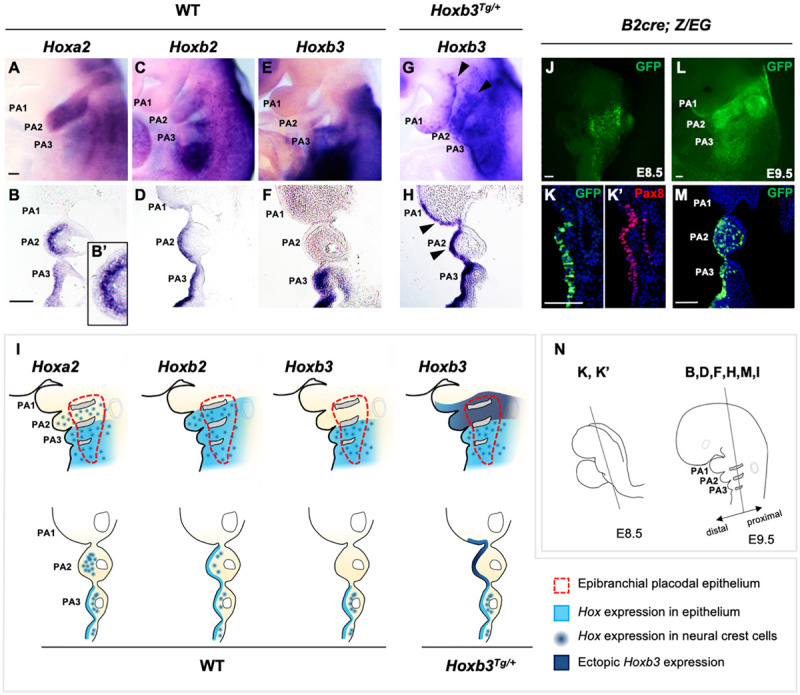
Expression of *Hox* genes in pharyngeal arches of wildtype and *Hoxb3^*Tg*/+^* embryos. **(A–H)** Wholemount *in situ* hybridization and coronal sections showing expression of *Hoxa2, Hoxb2*, and *Hoxb3* in wildtype and mutant E9.5 embryos (*n* = 3). An enlarged image in **(B’)** showing the absence of *Hoxa2* expression in the epithelium covering PA2. Arrowheads in **(G,H)** indicate ectopic expression of *Hoxb3* in the pharyngeal epithelium of PA1 and PA2. **(I)** Schematic summary of *Hox* gene expression in the pharyngeal ectoderm, epibranchial placodal region, and neural crest cells of E9.5 wildtype and *Hoxb3*^*Tg/+*^ embryos. **(J,K)** Wholemount GFP autofluorescence **(J)** and co-immunostaining of GFP **(K)** and Pax8 **(K’)** in coronal section of E8.5 *B2-Cre;Z/EG* embryos (*n* = 3). **(L,M)** Wholemount GFP autofluorescence **(L)** and immunostaining of GFP in coronal section **(M)** of E9.5 *B2-Cre; Z/EG* embryos (*n* = 3). **(N)** Schematic diagram illustrating the plane of embryo sections for the indicated panels in this figure. PA1, PA2, and PA3 indicate the 1st, 2nd, and 3rd pharyngeal arches. Scale bars: 100 μm.

The expression of *Hox* genes in the developing PAs have been well characterized. However, the expression and function of *Hox* genes in the pharyngeal surface ectoderm and the epibranchial placodal epithelium of the pharyngeal arches are not well understood. We showed that *Hoxa2* and *Hoxb2* were both expressed in PA2, PA3 and posterior PAs, and *Hoxb3* was expressed in PA3 and posterior PAs at E9.5 (Figures 1A,C,E). Interestingly, coronal sections at the level of proximal pharyngeal arches (refer to Figure 1N) of embryos hybridized with *Hoxa2* and *Hoxb2* probes revealed differential expression of these two genes in PA2. *Hoxa2* was expressed strongly in the neural crest derived ectomesenchyme but hardly detectable in the epibranchial placodal epithelium covering PA2 (Figures 1B,B’), although it was expressed in placodal epithelium and neural crest cells of posterior PAs. On the contrary, *Hoxb2* was distinctly expressed in the epibranchial placodal epithelium of PA2 and posterior PAs (Figure 1D), while expressed at lower levels in the neural crest cells of these PAs (Figure 1D). *Hoxb3* was strongly expressed in the pharyngeal surface ectoderm (Figure 1E), epibranchial placodal epithelium and neural crest cells of PA3 and posterior PAs (Figure 1F).

We have previously demonstrated that in the developing PAs, the proximal pharyngeal epithelium was derived from the posterior placodal area and overlap with the epibranchial placodal cells (Figure 1I, red dotted line; Zhang et al., 2017; Wang et al., 2020), while the distal PAs were enveloped by pharyngeal ectodermal epithelium. Therefore, the proximal and distal pharyngeal epithelium were composed of developmentally distinct epithelial cells. The *Hox* genes delineate the antero-posterior identity of the pharyngeal arches, including the neural crest cells delaminated from specific hindbrain rhombomeres. Neural crest cells in PA2 expressed *Hoxa2* and *Hoxb2*, while those in PA3 and posterior PAs expressed *Hoxb3* in addition to *Hoxa2* and *Hoxb2*. However, for the pharyngeal epithelium, we showed that *Hoxb2* marked PA2 (Figures 1D,I) and *Hoxb3* marked PA3 (Figures 1F,I). The distinct expression patterns of *Hox* genes in both the proximal and distal pharyngeal epithelium of specific PAs suggest a role of these genes in maintaining the functions of epibranchial placodal cells in the proximal regions.

### Ectopic Expression of *Hoxb3* in PA2 of *Hoxb3^*Tg*/+^* Transgenic Mutants

To investigate the role of the combinatorial *Hox* code in PA development as well as to understand the potential interactions between placodal cells and underlying neural crest cells, we investigated the *Hoxb3^*Tg*/+^* gain-of-function transgenic mutant generated with the *Hoxb2-r4* enhancer (Wong et al., 2011). The *Hoxb2-r4* enhancer could direct reporter gene expression in hindbrain rhombomere 4 and PA2 (Maconochie et al., 1997; Ferretti et al., 2000). We analyzed the enhancer activity in the pharyngeal epithelium using the *B2-Cre* [*Hoxb2-r4-Cre*; (Szeto et al., 2009)] mouse line. Wholemount *B2-Cre;Z/EG* embryos showed expression of the GFP reporter in the pharyngeal region at E8.5 (Figure 1J), and in PA2 at E9.5 (Figure 1L). By immunostaining with Pax8, one of the earliest markers of the posterior placodal area (Schlosser and Ahrens, 2004; Washausen and Knabe, 2017), on coronal sections of E8.5 embryo, we showed that GFP + cells also expressed Pax8 (Figures 1K,K’), confirming the enhancer activity in the placodal epithelial cells. Coronal sections of E9.5 embryos showed *B2-Cre* activity in the proximal placodal epithelium and pharyngeal ectodermal region from second to posterior arches, as well as the neural crest cells in PA2 and PA3 (Figures 1L,M).

In the *Hoxb3*^*Tg/+*^ mutant, driven by the *Hoxb2-r4* enhancer element, ectopic *Hoxb3* was expressed not only in the entire proximal and distal PA2, but also in posterior region of PA1 (Figures 1G,H). Coronal sections of *Hoxb3*^*Tg/+*^ embryos showed that ectopic expression of *Hoxb3* was restricted to the pharyngeal epithelium of posterior PA1 and PA2 (Figure 1H), no expression could be observed in the neural crest cells of PA2. Therefore, in the *Hoxb3^*Tg*/+^* mutant, we have ectopically expressed *Hoxb3* specifically in the pharyngeal epithelium of PA2 and posterior PA1, providing a gain-of-function genetic condition for analysis of epithelial cell function during PA development.

### Ectopic Expression of *Jag1* in PA2 Epithelium of *Hoxb3*^*Tg/+*^ Mutant

**FIGURE 2:**
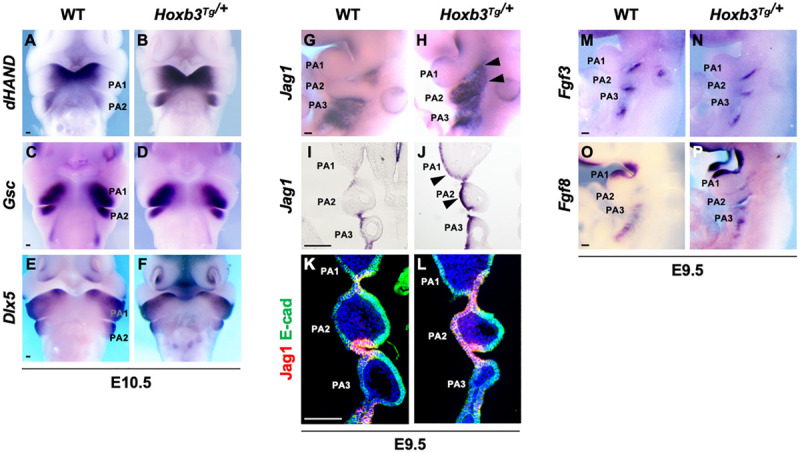
Pharyngeal arch patterning and ectopic expression of Jag1 in the *Hoxb3*^*Tg/+*^ mutants. **(A–F)** Wholemount *in situ* hybridization showing expression of *dHAND*, *Gsc*, and *Dlx5* in wildtype and *Hoxb3*^*Tg/+*^ embryos at E10.5 (*n* > 3). **(G,H)** Wholemount *in situ* hybridization of *Jag1* showing ectopic expression in PA1 and PA2 (arrowheads) of E9.5 *Hoxb3*^*Tg/+*^ embryos (*n* = 4). **(I,J)** Coronal sections of embryos subjected to wholemount *in situ* hybridization of *Jag1* showing ectopic expression in the ectodermal epithelium of PA1 and PA2 (arrowheads) in *Hoxb3*^*Tg/+*^ embryos (*n* = 3). **(K,L)** Immunostaining for Jag1 (red) and E-cadherin (green) on coronal sections of E9.5 wildtype and *Hoxb3*^*Tg/+*^ embryos (*n* = 5). **(M–P)** Wholemount *in situ* hybridization of *Fgf3* and *Fgf8* on E9.5 wildtype and *Hoxb3*^*Tg/+*^ embryos (*n* > 3). PA1, PA2, and PA3 indicate the 1st, 2nd, and 3rd pharyngeal arches. Scale bars: 100 μm.

To investigate the consequences of ectopic expression of *Hoxb3* in pharyngeal arch patterning, we examined the expression of genes in the distal PAs including *dHAND* which marked the medial regions (Figure 2A), *Gsc* which marked the ventral aspects (Figure 2C), and *Dlx5* which marked the lateral regions (Figure 2E) of PA1 and PA2 in E10.5 embryos. However, the regional specific expression patterns of *dHAND, Gsc*, and *Dlx3* were not changed in the *Hoxb3*^*Tg/+*^ mutants (Figures 2B,D,F), indicating the patterning of distal PAs remained unaffected with the ectopic expression of *Hoxb3*.

As Notch signaling has been shown to be critical for the development of epibranchial placodal epithelium and proximal PAs (Zhang et al., 2017; Wang et al., 2020), we examined *Jag1* which was expressed in the proximal pharyngeal epithelium of developing PAs and restricted to the pharyngeal clefts and pouches as the segmental plates were formed at E9.5 (Figures 2G,I,K). Interestingly, we found that the expression of *Jag1* was ectopically activated in the entire pharyngeal ectodermal region of PA2 and posterior PA1 in the *Hoxb3*^*Tg/+*^ mutants at E9.5 (Figures 2H,J). The expression of *Jag1* in the epibranchial placodal epithelium of PA1 and PA2 as shown in coronal section of *Hoxb3*^*Tg/+*^ mutant (Figure 2J) was remarkably similar to the ectopic *Hoxb3* expression domains (Figure 1H). By immunostaining we could detect Jag1 protein in the pharyngeal epithelium of PA2 of *Hoxb3*^*Tg/+*^ mutant (Figure 2L), suggesting that ectopic expression of *Hoxb3* in the pharyngeal epithelium in PA2 could activate Jag1 protein expression in PA2.

We have previously shown that over-expression of the Notch receptor protein N1-ICD in the epibranchial placodal epithelium would lead to misexpression of Fgf ligands and cell fate changes (Wang et al., 2020). Therefore, we examined the expression of *Fgf3* and *Fgf8*, which marked placodal epithelial cells flanking the pharyngeal clefts (Figures 2M,O). However, we found that ectopic expression of the Notch ligand *Jag1* did not cause changes of *Fgf3* or *Fgf8* expression in *Hoxb3****^*Tg/+*^*** mutants (Figures 2N,P), suggesting that the pharyngeal epithelial cell fates were not affected.

### Malformations of Neural Crest Derived Structures in the *Hoxb3*^*Tg*^ Mutants

**FIGURE 3:**
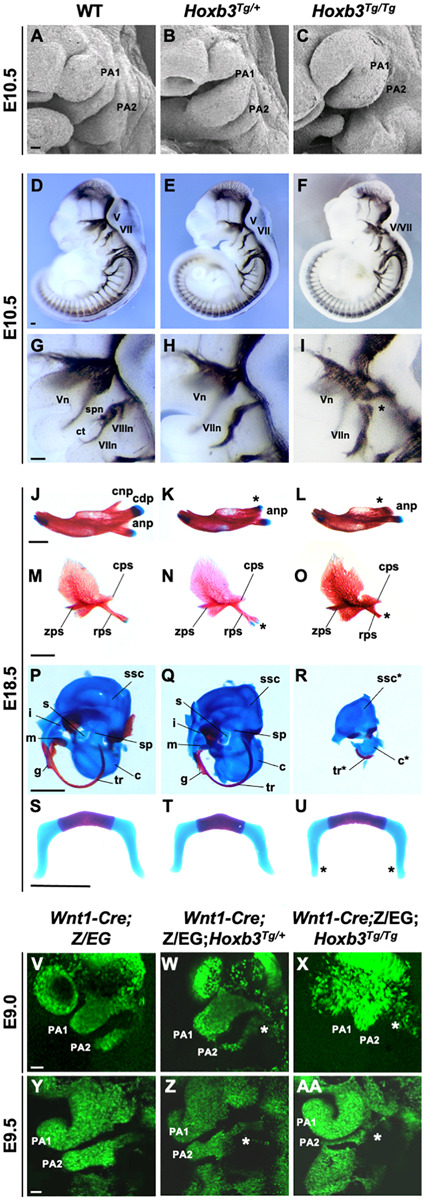
Hypoplastic PA2 and defective neural crest development in *Hoxb3*^*Tg*^ mutants. **(A–C)** Scanning electron microscopy of E10.5 wildtype, *Hoxb3*^*Tg/+*^ and *Hoxb3*^*Tg/Tg*^ embryos (*n* = 3). **(D–I)** Neurofilament antibody (2H3) staining showing cranial nerve defects in wildtype and *Hoxb3*^*Tg*^ mutants at E10.5 (*n* > 3). **(G–I)** are enlargements of pharyngeal regions showing abnormal facial nerve branching in *Hoxb3*^*Tg/+*^ and nerve fusion in *Hoxb3*^*Tg/Tg*^ (indicated with asterisk). V/Vn, trigeminal nerve; VII/VIIn, facial nerve; VIIIn, vestibuloaoustic nerve; spn, superficial petrosal nerve; and ct, chorda tympani nerve. **(J–R)** Skeletal preparations showing defective PA1 and PA2 neural crest cell derived structures in the *Hoxb3*^*Tg*^ mutants (*n* > 5). **(J–L)** Skeletal preparations showing defective mandibles of *Hoxb3*^*Tg*^ mutants. Absence of condylar process indicated with asterisks. **(M–O)** Defective squamous bones in *Hoxb3*^*Tg*^ mutants. Abnormal retrotympanic process indicated with asterisks. anp, angular process, cdp and cnp, condylar and coronoid processes of mandible; cps, caudal process of squamous; rps, retrotympanic process of squamous; zps, zygomatic process of squamous; and g, gonial. **(P–R)** Defective middle ear bones, inner ear otic capsules and associated elements in *Hoxb3*^*Tg*^ mutants. c, cochlea; i, incus; m, malleus; s, stapes; sp, styloid process; ssc, semi-circular canals; and tr, tympanic ring. **(S–U)** Skeletal preparations showing mild defects in the hyoid cartilage of *Hoxb3*^*Tg/Tg*^ mutants. *indicates the greater horn. **(V–AA)** Wholemount GFP autofluorescence of *Wnt1-Cre;Z/EG;Hoxb3^*Tg*^* compound mutant embryos showing abnormal distribution of GFP-positive neural crest cells (asterisks) to PA2 in E9.0 and E9.5 *Hoxb3*^*Tg/+*^ and *Hoxb3*^*Tg/Tg*^ embryos. PA1 and PA2 indicate the 1st and 2nd pharyngeal arches (*n* > 3). Scale bars: A-I and V-AA, 100 μm; J-U, 1,000 μm.

We first examined the phenotypes of the *Hoxb3*^*Tg*^ mutant PAs by scanning electron microscopy. Although the pharyngeal arch phenotypes of the mutant were mild, it was evident that the PA2 was smaller in *Hoxb3*^*Tg/+*^ and significantly reduced in size in the *Hoxb3*^*Tg/Tg*^ at E10.5 (Figures 3A–C). To investigate the neural components in the pharyngeal region, wholemount immunohistochemistry was performed using 2H3 anti-neurofilament antibody. In the *Hoxb3*^*Tg/+*^ mutants, the vestibulocochlear nerve (VIIIn), the superficial petrosal nerve (spn), and the chorda tympanic nerve (ct) were absent (Figures 3D,E,G,H). In the *Hoxb3*^*Tg/Tg*^ mutants, there was abnormal fusion between the trigeminal ganglion (V) and the facial acoustic ganglia (VII; Figures 3F,I).

To examine the phenotypes of neural crest derived skeletal components, neonates were examined by skeletal staining. Among the PA1 derived structures, the mandible was reduced in length and poorly ossified and the coronoid process was absent in both *Hoxb3*^*Tg/+*^ and *Hoxb3*^*Tg/Tg*^ mutants. The condylar process was present in the *Hoxb3*^*Tg/+*^ embryos, but missing in the *Hoxb3*^*Tg/Tg*^ embryos (Figures 3J–L). The maxillary arch derived squamous bone was slightly affected with a duplication found in the retrotympanic process in the *Hoxb3*^*Tg/+*^ embryos (Figures 3M,N). The squamous bone was more severely affected in the *Hoxb3*^*Tg/Tg*^ mutants (Figure 3O). Among the PA1 derived middle ear structures, the malleus and incus were hypoplastic in the *Hoxb3*^*Tg/+*^ embryos (Figures 3P,Q), and absent in the *Hoxb3*^*Tg/Tg*^ mutants (Figure 3R). The otic capsule and tympanic ring were hypoplastic in the *Hoxb3*^*Tg/Tg*^ mutants (Figure 3R). For the PA2 derived skeletal elements, the styloid process was slightly truncated in *Hoxb3*^*Tg/+*^, while both the stapes and styloid process were severely reduced in the *Hoxb3*^*Tg/Tg*^ mutants (Figures 3Q,R). The hyoid cartilage appeared normal in *Hoxb3*^*Tg/+*^, the morphology of the greater horn of the hyoid cartilage was mildly affected in the *Hoxb3*^*Tg/Tg*^ mutants (Figures 3S–U). Taken together, we showed that the development of both PA1 and PA2 neural crest derived skeletal structures were affected in the *Hoxb3*^*Tg*^ transgenic mutants.

### Deficient Neural Crest Cell Migration Into PA2 of *Hoxb3^*Tg*^* Mutant

We hypothesized that the hypoplastic PA2 and abnormal neural crest derived structures in the *Hoxb3*^*Tg*^ mutants could be due to neural crest cell deficiencies. By using the *Wnt1-cre*;*Z/EG* mice as neural crest cell lineage marker, we generated compound mutants and traced the migrating neural crest cells at E9.0 and E9.5. We observed consistently fewer neural crest cells migrating to PA2 of the *Hoxb3*^*Tg/+*^, and significantly few cells entering PA2 of the *Hoxb3*^*Tg/Tg*^ mutants at E9.0 and E9.5 (Figures 3V–AA). Therefore, the multiple neural and skeletal phenotypes observed in the mutant pharyngeal regions were likely due to insufficient neural crest cells populating PA2.

### *Hoxb3* Activates the Expression of Jag1 by Direct Binding to Specific Genomic Sites

**FIGURE 4:**
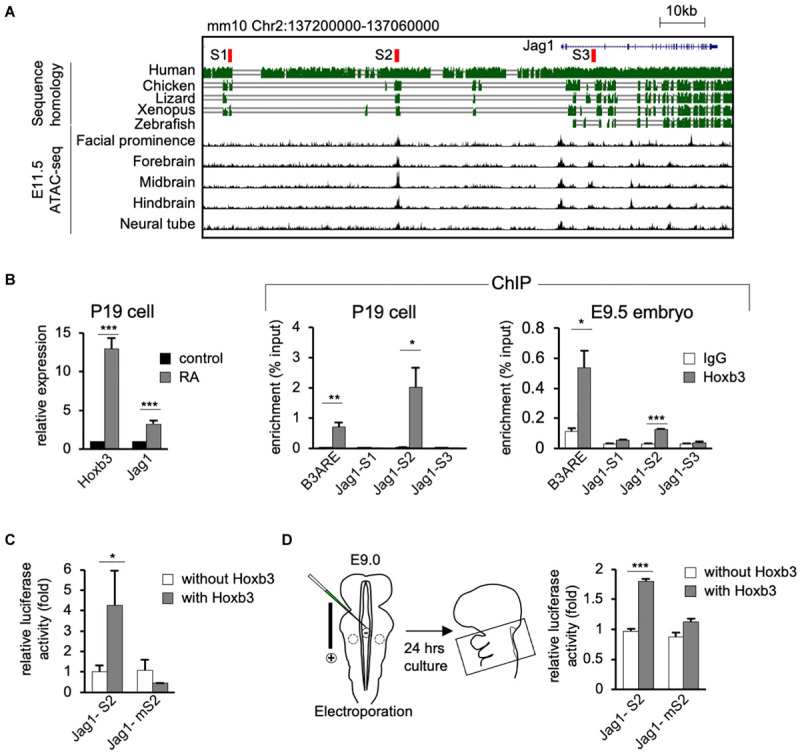
Hoxb3 regulates Jag1 expression by binding to *cis*-regulatory region S2. **(A)** Genome browser tracks showing mouse *Jag1* locus including 100 Kb upstream genomic region. Alignment of genomic regions of human, chicken, lizard and xenopus revealed conserved regions and identification of potential Hoxb3 binding regions S1, S2, and S3 (highlighted in red). ATAC-seq tracks from ENCODE database of multiple E11.5 mouse embryo tissues showed an open chromatin region (S2) 40 kb upstream of *Jag1* transcription start site. **(B)** *In vitro* and *in vivo* chromatin immunoprecipitation (ChIP) analyses using RA treated P19 cells and mouse embryos. qRT-PCR analysis of P19 cells showed elevated expression of *Hoxb3* and *Jag1* after RA treatment. ChIP qRT-PCR analysis showed that Hoxb3 could bind to S2 site of *Jag1*, but not S1 or S3 sites. Hoxb3 could also bind to the B3ARE site, an autoregulatory element which served as a positive control. ChIP qRT-PCR analysis using E9.5 embryos also showed that Hoxb3 could bind to the S2 site and the positive control B3ARE site. **(C)** *In vitro* luciferase reporter assay using 293T cells. Expression of *Hoxb3* could activate luciferase activity when co-transfected with *Jag1-S2* reporter, the activity was much lower when co-transfected with *Jag1-mS2* reporter containing mutated S2 site. **(D)** *Ex vivo* luciferase reporter assay using E9.0 mouse embryos. Co-electroporation of *Hoxb3* expression vector with *Jag1-S2* or *Jag1-mS2* luciferase reporter showed that *Hoxb3* could elevate luciferase activity via the normal S2 site. Data in **(B–D)** are shown as mean ± SE (*n* = 3 biological replicates). *P < 0.05, **P < 0.01, and ***P < 0.001.

To investigate the possibility that *Hoxb3* may directly activate *Jag1* expression by binding to genomic regulatory elements, we performed bioinformatics analyses to identify possible Hoxb3 binding sites around the *Jag1* gene. Using the consensus Hoxb3 binding matrix from JASPAR database, we searched the conserved regions within 100 kb upstream of Jag1 among six vertebrate species including mouse, human, chicken, lizard, Xenopus, and zebrafish. Furthermore, by comparing the open chromatin regions upstream of *Jag1* from several E11.5 mouse tissues, we identified three potential regulatory sites, named S1, S2, and S3. The S1 and S2 sites were located upstream of *Jag1* gene (mm10 chr2:137190579-137190732; chr2:137153318-137153475), the S3 site was in the intron between Exon2 and Exon3 of *Jag1* (mm10 chr2:137110522-137110680; Figure 4A).

To test whether Hoxb3 can directly bind to the three identified binding sites around *Jag1* gene, we performed *in vitro* and *in vivo* chromatin immunoprecipitation assays using P19 cells and mouse embryos. We treated P19 cells with RA to activate the endogenous expression of *Hoxb3*. The Quantitative real-time PCR (qRT-PCR) results showed that the expression of *Hoxb3* and *Jag1* were significantly increased after RA treatment (Figure 4B). Chromatin fragments from RA treated P19 cells were immunoprecipitated with a Hoxb3 antibody (Supplementary Figure 1). Several sets of primers were used to detect potential binding to the S1, S2, and S3 sites. A known Hoxb3 binding site, B3ARE in the auto-regulatory element (Yau et al., 2002; Wong et al., 2011), was used as a positive control. The qRT-PCR results of ChIP assays showed that Hoxb3 antibody could immunoprecipitate the B3ARE and S2 sites but not S1 or S3 sites. We further performed *in vivo* ChIP assays using wildtype E9.5 embryos. The entire embryos were lysed and the chromatin fragments were immunoprecipitated with IgG and Hoxb3 antibodies. The PCR results showed that endogenous Hoxb3 protein could form complex with chromatin fragments containing the S2 binding site (Figure 4B). To further investigate whether Hoxb3 could positively regulate the expression of *Jag1* through binding to the S2 site, we performed luciferase assay on human embryonic kidney cells 293T. Luciferase activity was significantly increased in the presence of Hoxb3, but no activation could be observed when Jag-S2 site was mutated (Figure 4C). To further investigate whether Hoxb3 could positively regulate the expression of *Jag1* through binding to the S2 site, we performed mouse *ex vivo* electroporation experiment (Figure 4D). Co-expression of the luciferase reporter containing S2 binding site of *Jag1* (Jag1-S2) and *Hoxb3* expression vector, luciferase activity could be detected in the embryo lysates. Without Hoxb3, luciferase activity was significantly reduced. However, with the S2 site mutated in the *Jag1* luciferase reporter (Jag1-mS2), electroporation of Hoxb3 expression vector could not increase the luciferase activity.

In conclusion, by molecular studies we demonstrated that endogenous Hoxb3 protein could directly activate the expression of *Jag1* through specific binding to the S2 regulatory site. This study revealed a novel regulatory mechanism for *Jag1* gene expression. During normal development, cell type-specific expression of *Jag1* could be regulated by *Hoxb3.*

## Discussion

The roles of *Hox* genes in providing positional information in axial development have long been established. In the pharyngeal region, combinatorial *Hox* expression defines the positional identify of the hindbrain rhombomeres and associated neural crest cells. It has been suggested that the surface ectoderm also expresses combinatorial *Hox* genes, presenting segmentally patterned “ectomeres” where the ectodermal epithelium is regionally specified and associated with the *Hox*-patterned pharyngeal arches (Couly and Le Douarin, 1990; Hunt et al., 1991). However, the antero-posterior segmental specific functions of the ectodermal pharyngeal epithelium in the PAs remain elusive. The pharyngeal epithelium is important for providing environment cues for cranial neural crest cell homing, survival and differentiation. Our gain-of-function *Hoxb3*^*Tg*^ mutant analysis here suggests that the cross-talk of *Hox* genes with molecular signaling in the pharyngeal epithelium is required for interaction and maintenance of neural crest cells during PA development.

### Expression of *Hox* Genes and Neural Crest Defects in *Hoxb3*^*Tg*^ Mutant PAs

In the *Hoxb3^*Tg*^* mutant, we have previously shown that hindbrain neurogenesis defect was observed as a result of the loss of *Hoxb1* expression in hindbrain r4 (Wong et al., 2011). However, here we found that the generation, delamination and migration of r4-derived neural crest was not affected, except that the population of neural crest cells entering the mutant PA2 were reduced (Figures 3W,X,Z,AA). The expression patterns of *Hoxa1, Hoxa2*, and *Hoxb1* in the PAs were not changed in the *Hoxb3*^*Tg/+*^ mutant (Supplementary Figure 2). Therefore, in the *Hoxb3*^*Tg/+*^ mutant, normal combinatorial *Hox* genes were expressed in the neural crest cells. The dorso-ventral patterning of PAs also remained unaffected in the mutant (Figures 3A–F). The specific changes in the *Hoxb3*^*Tg/+*^ mutant were with the surface ectoderm, the entire proximal-distal pharyngeal epithelium of PA2 and posterior PA1 were the only cells expressing *Hoxb3* ectopically. Although PA1 is normally a *Hox*-free zone, and the enhancer activity of the *Hoxb2-r4* element used to drive ectopic gene expression is expected to cover PA2 only (Figures 1L,M), as a result of transgene insertion activity, ectopic *Hoxb3* expression was unexpectedly also found in PA1 epithelium. The ectopic expression patterns of *Hoxb3* would explain that neural crest structures derived from both PA1 and PA2 were affected in the *Hoxb3*^*Tg*^ mutants. The malformations could be a direct effect of ectopic *Hoxb3* expression in the ectodermal pharyngeal epithelium, or through other signaling molecules including *Jag1* as discussed below.

In the heterozygous *Hoxb3*^*Tg/+*^ transgenic mutant, the PA phenotypes were mild, but the hypoplasia of PA2 and craniofacial malformations were more evident in the homozygous *Hoxb3*^*Tg/Tg*^ with a higher dosage of the transgene. The fusion of the trigeminal and facioacoustic ganglia observed in *Hoxb3*^*Tg/Tg*^ suggests that there might be mixing or mis-routing of neural crest cells. As visualized by *Wnt1-Cre;Z/EG* lineage tracing experiments, in E9.0 *Hoxb3*^*Tg/Tg*^ mutant embryo the neural crest cells appeared aggregated around the proximal PA1 and PA2 region (Figure 3X). As many of the neural crest cells did not reach PA2, a deficiency of neural crest cells would explain the pharyngeal skeletal phenotype in the *Hoxb3*^*Tg*^ mutants.

### *Hoxb3* Directly Regulates *Jag1* Expression

To address how ectopic *Hoxb3* expression in the pharyngeal epithelium could affect interaction with neural crest cells, we examined the Notch signaling ligand *Jag1* and found that *Hoxb3* could directly regulate *Jag1* expression. *Jag1* is required for craniofacial morphogenesis and other developmental processes (Xue et al., 1999; Humphreys et al., 2012), but the spatial-temporal regulation of *Jag1* expression during development is unclear. By bioinformatics analyses, we identified several Hoxb3 binding sites in the highly conserved genomic regions around *Jag1* gene. Using *in vivo* ChIP assays and *in vitro*/*ex vivo* luciferase reporter assays, we demonstrated that Hoxb3 could directly bind to the S2 site and *trans*-activate gene expression. From ENCODE databases, many other transcription factors binding sites could be identified around S2, indicating that this 40 Kb upstream genomic region could be a *cis*-regulatory enhancer element for activating *Jag1* expression. In the *Hoxb3*^*Tg*^ gain-of-function mutant, we showed that *Jag1* expression could be specifically activated by ectopic *Hoxb3* expression in the pharyngeal epithelium of PA1 and PA2 (Figure 2J). It is possible that during normal craniofacial development, *Hoxb3* is also required to maintain *Jag1* expression in the posterior pharyngeal arches and clefts to coordinate with the pharyngeal segmentation process.

The expression of *Jag1* has been reported to be activated by other *Hox3* genes. In hemogenic endothelium, overexpression of *Hoxa3* could activate the expression of *Jag1* (Sanghez et al., 2017). In HEL and K563 cells, overexpression of *Hoxd3* also led to elevated *Jag1* expression. Moreover, the expression level of *Jag1* was dependent on transfected *Hoxd3* expression (Taniguchi et al., 2001). These studies confirmed that the *Hox3* genes could regulate *Jag1* expression and Notch signaling in broader developmental contexts.

### The Role of *Jag1* in PA Development

Recent studies of the proximal PA and epibranchial placode suggest that pharyngeal segments are gateways for neural crest cell migration into the PAs. Entering between the pharyngeal clefts, the neural crest cells populate the PAs distally, where subsequent morphogenetic events take place. For example, *Sox3* knockout mice with defective epibranchial placodal cells in the proximal pharyngeal epithelium displayed mis-migration of neural crest cells into the PAs and resulted in cranial skeletal defects (Rizzoti and Lovell-Badge, 2007). On the other hand, in the *Eya1* mutant which failed to maintain the epibranchial placodal cells, defective neural crest-derived maxilla and mandible were observed (Xu et al., 1999; Zhang et al., 2017). Importantly, both *Sox3* and *Eya1* are not expressed in neural crest but in the epibranchial placodal cells, indicating a non-cell autonomous role of epibranchial placodal epithelium in controlling the migration of neural crest cells to the PAs. In the *Hoxb3*^*Tg/+*^ mutants described in this study, ectopic expression of *Hoxb3* and *Jag1* were found only in the pharyngeal epithelium, the neural crest-derived skeletal defects could be the consequence of misexpression of *Hoxb3* in the epibranchial placodal cells in the proximal PA.

**FIGURE 5:**
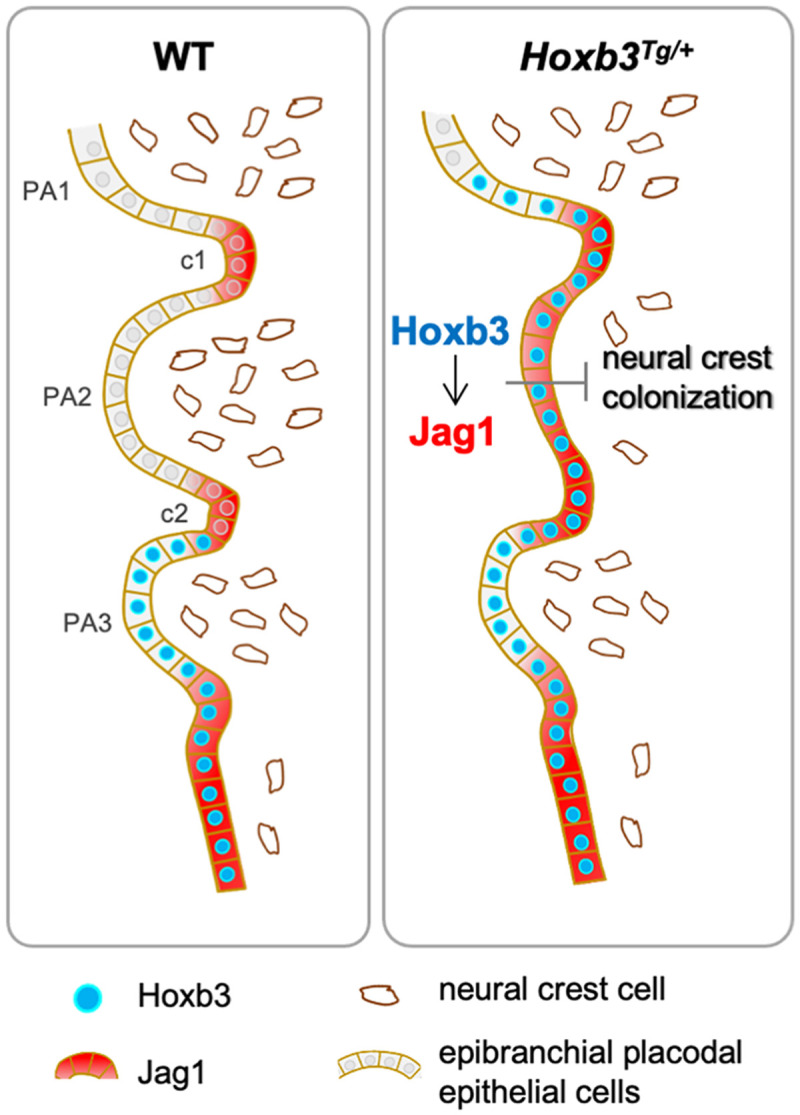
Hoxb3 regulate Jag1 expression and affect pharyngeal epithelium and neural crest interactions. Schematic diagram showing expression of *Hoxb3* and *Jag1* in epibranchial placodal epithelial cells of wildtype and *Hoxb3*^*Tg*^ mutant. In wildtype embryos, *Jag1* expression is restricted to the pharyngeal clefts which function as boundaries for neural crest migration into the pharyngeal arches. In the *Hoxb3*^*Tg*^ mutant, ectopic *Hoxb3* expressed in the epibranchial placodal epithelium of PA2 could activate *Jag1* expression directly. Elevated *Jag1* expression is incompatible with neural crest migration into the pharyngeal arch. As a result, deficiency of neural crest colonization into PA2 led to abnormal development of multiple neural crest derivatives in the pharyngeal region of the *Hoxb3*^*Tg*^ mutant. PA1, PA2, and PA3 indicate the 1st, 2nd, and 3rd pharyngeal arches; c1 and c2 indicate the 1st and 2nd pharyngeal clefts.

We have previously shown that *Jag1* is initially broadly expressed in the proximal pharyngeal region in the epibranchial placodal cells at early stage (E8.5), but its expression is down-regulated in PA epithelium and became restricted to the pharyngeal clefts at around E9.5 (Zhang et al., 2017). While the functional role of *Jag1* in pharyngeal segmentation is not known, it appears that *Jag1* expressing regions serve as boundaries for neural crest cell migration. In the *Hoxb3*^*Tg/+*^ mutant, persisted expression of *Jag1* in the proximal pharyngeal epithelium of PA2 could inhibit the colonization of neural crest cells into the arch (Figure 5), leading to the craniofacial abnormalities. Our genetic study has revealed the importance of the pharyngeal epithelial cells in interacting with cranial neural crest for proper craniofacial development.

## Data Availability Statement

The original contributions presented in the study are included in the article/Supplementary Material, further inquiries can be directed to the corresponding author.

## Ethics Statement

The animal study was reviewed and approved by The University of Hong Kong Committee on the Use of Live Animals for Teaching and Research (CULATR Nos. 4357–17 and 4588–18).

## Author Contributions

HZ, EW, and MS conceived the study, designed the experiments, and wrote the manuscript. HZ, JX, KS, KT, JS-P, LW, EW, and WC conducted the experiments. ST, JS-P, EW, and HZ managed the experimental animals. JX, KS, KT, and MS reviewed and revised the manuscript and edited the figures. All authors participated in data analysis and revisions of the manuscript. MS obtained resources and funding for the study.

## Conflict of Interest

The authors declare that the research was conducted in the absence of any commercial or financial relationships that could be construed as a potential conflict of interest.
