# Supplementary figures and images for: Hoxb3 Regulates Jag1 Expression in Pharyngeal Epithelium and Affects Interaction With Neural Crest Cells
Source: Front Physiol. 2021 Jan 11;11:612230. doi: 10.3389/fphys.2020.612230 (PMC7830521; doi:10.3389/fphys.2020.612230)

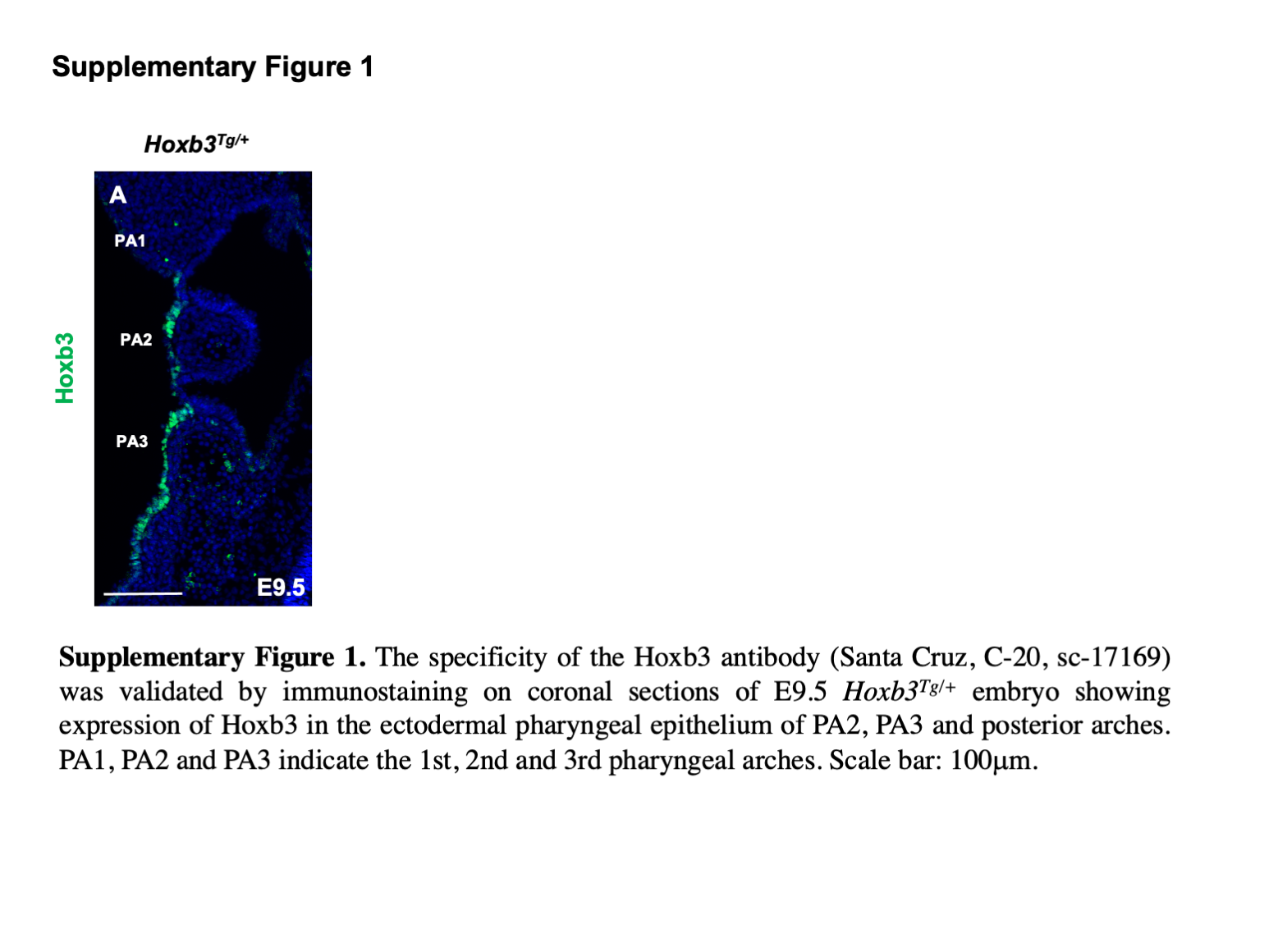

Supplement: Supplementary file 1 [file Image_1.tif]

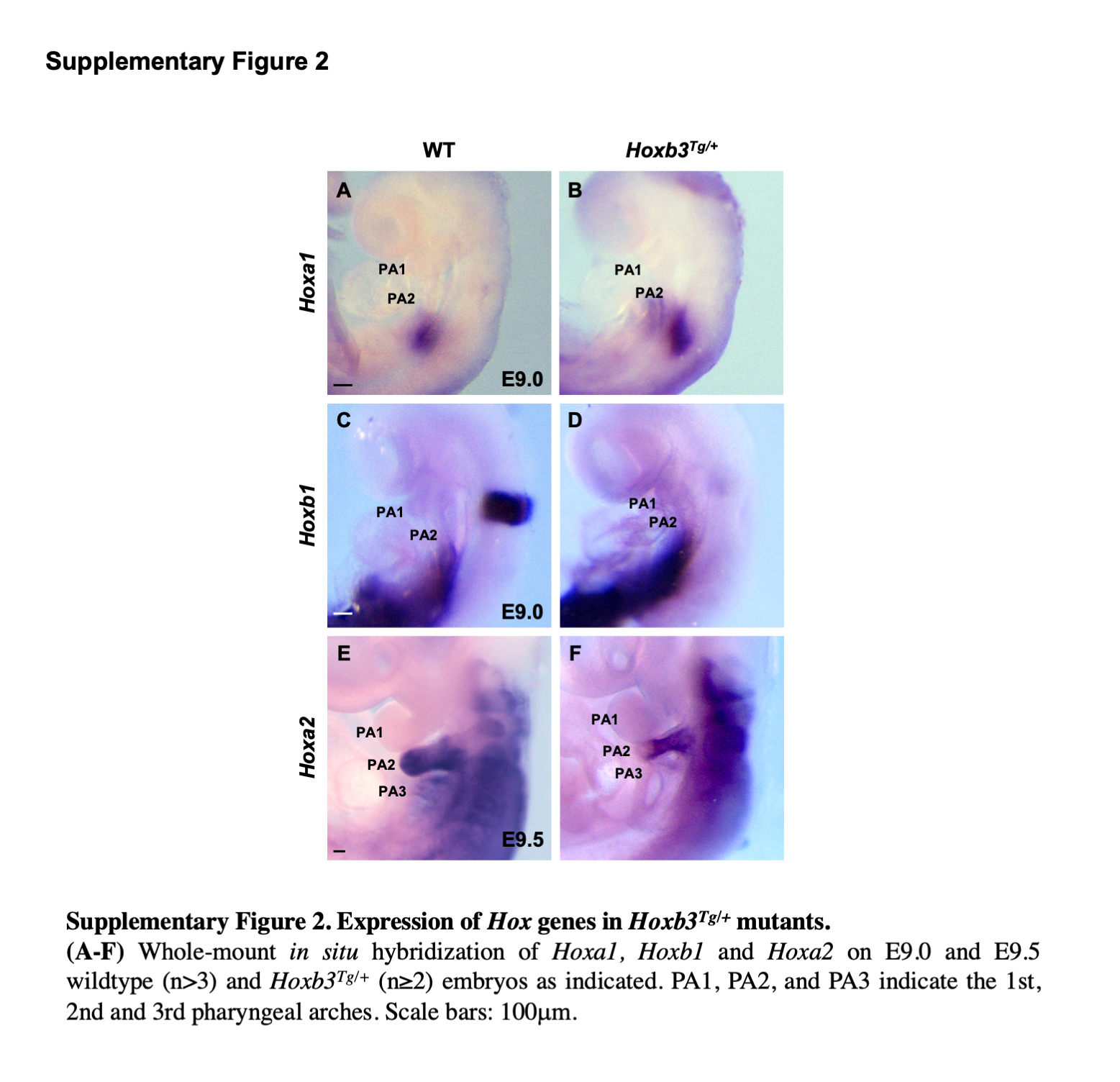

Supplement: Supplementary file 2 [file Image_2.tif]
